# Trefoil Factor 3 as an Endocrine Neuroprotective Factor from the Liver in Experimental Cerebral Ischemia/Reperfusion Injury

**DOI:** 10.1371/journal.pone.0077732

**Published:** 2013-10-18

**Authors:** Shu Q. Liu, Derek Roberts, Brian Zhang, Yupeng Ren, Li-Qun Zhang, Yu H. Wu

**Affiliations:** 1 Biomedical Engineering Department, Northwestern University, Evanston, Illinois, United States of America; 2 Rehabilitation Institute of Chicago, Chicago, Illinois, United States of America; Universidade de Sao Paulo, Brazil

## Abstract

Cerebral ischemia, while causing neuronal injury, can activate innate neuroprotective mechanisms, minimizing neuronal death. In this report, we demonstrate that experimental cerebral ischemia/reperfusion injury in the mouse causes upregulation of the secretory protein trefoil factor 3 (TFF3) in the hepatocyte in association with an increase in serum TFF3. Partial hepatectomy (~60% liver resection) immediately following cerebral injury significantly lowered the serum level of TFF3, suggesting a contribution of the liver to the elevation of serum TFF3. Compared to wild-type mice, TFF3^-/-^ mice exhibited a significantly higher activity of caspase 3 and level of cell death in the ischemic cerebral lesion, a larger fraction of cerebral infarcts, and a smaller fraction of the injured cerebral hemisphere, accompanied by severer forelimb motor deficits. Intravenous administration of recombinant TFF3 reversed changes in cerebral injury and forelimb motor function due to TFF3 deficiency. These observations suggest an endocrine neuroprotective mechanism involving TFF3 from the liver in experimental cerebral ischemia/reperfusion injury.

## Introduction

Cerebral ischemia, while causing neuronal injury and neurological deﬁcits, can activate innate neuroprotective mechanisms, minimizing neuronal death and improving cerebral function. Such mechanisms are implemented by upregulation and/or release of protective factors from the injured neurons, activated glial cells, and/or leukocytes recruited to the ischemic brain tissue. Recognized neuroprotective factors include, but are not limited to, adenosine [1,2], Gamma-aminobutyric acid (GABA) [1,3-5], opioids [6,7], interleukin 1α (IL1α)/IL1β [8], IL6 [9-13], leukemia inhibitory factor (LIF) [12], erythropoietin [14,15], brain-derived neurotrophic factor (BDNF) [16,17], nerve growth factor (NGF) [17], transforming growth factor β (TGFβ) [18-24], and vascular endothelial growth factor (VEGF) [25]. These factors can interact with cognate receptors, activate cell survival signaling networks, and suppress injurious cell signaling events, thereby rescuing neurons from irreversible injury in the ischemic penumbra [1,25-27]. Furthermore, cerebral ischemia induces proliferation of neural stem and progenitor cells [28]. These cells can upregulate and release growth factors, including BDNF and VEGF, contributing to neuroprotection, neuronal regeneration, and angiogenesis in cerebral ischemia [25,28]. These previous investigations suggested the presence of naturally evolved neuroprotective mechanisms within the ischemic brain tissue, involving autocrine and paracrine factors [1,25]. In this report, we demonstrate a novel endocrine neuroprotective mechanism involving the secretory protein trefoil factor 3 (TFF3), which is upregulated in the liver in response to cerebral ischemia/reperfusion injury and released into the circulation, exerting a protective action against irreversible cerebral injury. 

In a multi-organ mammalian system, an inflammatory process involving leukocyte activation and cytokine secretion in an injured organ may cause systemic responses via the mediation of endocrine factors. Often, the systemic responses support the protection and repair processes of the injured organ [29-32]. We have recently demonstrated an endocrine cardioprotective mechanism in experimental myocardial ischemia involving upregulation of secretory proteins, including TFF3, in the liver [32]. These proteins are released into the circulation to protect penumbral ischemic myocardium from irreversible injury, minimizing myocardial infarction and improving myocardial function [32]. Given the similar pathological processes between experimental cerebral and myocardial ischemia, the liver may be activated in response to cerebral ischemia as well, contributing to cerebral protection. Here, we tested the hepatic response to experimental cerebral ischemia/reperfusion injury in the mouse, focusing on the expression of TFF3 in the hepatocyte, the contribution of the liver to the elevation of serum TFF3, and the neuroprotective action of TFF3. This investigation demonstrated the presence and significance of systemic neuroprotective mechanisms, establishing a new paradigm for cerebral ischemia research and development of neuroprotective therapeutics.

TFF3, also known as intestinal trefoil factor or ITF, is a ~7 kDa protein with 59 amino acids in the mature form and may be present as a monomer or dimer *in vivo* [33,34]. The human TFF3 gene is found on chromosome 21q22.3 [35] and the mouse TFF3 gene is found on chromosome 17 15.80 cM [36-38]. TFF3 is characterized by the presence of three intra-peptide disulfide bonds that result in the formation of a trefoil motif, a characteristic structure of TFF3 [35]. TFF3 was first identified from the mucus-secreting goblet cells of the small and large intestines [33,34] and has also been found in the hypothalamus and pituitary of the human [39] as well as the liver and urinary bladder of the mouse [40]. The primary function of TFF3 in the intestinal system is to support mucosal integrity under physiological conditions, protect the intestinal mucosa from mechanical and chemical insults, and facilitate mucosal healing after injury [41-49]. Experimental tests *in vitro* have demonstrated that TFF3 may enhance aggregation of intestinal mucin glycoproteins by forming inter-protein bridges. This reaction results in the formation of a stable viscous layer on the intestinal epithelium, protecting the epithelium from chemical and mechanical injury [33,34]. The TFF3 actions may be mediated by cognate receptor(s). Several investigations have demonstrated that TFF3 can bind a 28-kDa protein [50] and a 50 kDa glycoprotein [51] in intestinal epithelial cells, but these receptors have not been identified and characterized. To date, TFF3 has not been investigated for involvement in cerebral ischemia/reperfusion injury. 

## Materials and Methods

### Ethics Statement

All animal work was conducted according to the “Guide for the Care and Use of Laboratory Animals” by the National Research Council of the National Academies. The Northwestern University Institutional Animal Care and Use Committee approved all experimental procedures used in this investigation.

### Cerebral ischemia/reperfusion Injury

Cerebral ischemia/reperfusion injury was induced in TFF3^-/-^ and background-marched 129S1/SvImJ wild-type mice (both from Jackson Laboratory) by ligating the right middle cerebral artery and both common carotid arteries for 1 hr, a modified method based on previous reports [52,53]. Mice were anesthetized by intraperitoneal injection of ketamine (100 mg/kg) and xylazine (10 mg/kg of body weight). A skin incision was made between the right eye and the external auditory canal. A 2 mm hole was drilled on the temporal bone near the zygomatic arch. The middle cerebral artery was ligated with a silk suture thread at the first bifurcation above the zygomatic arch. Both common carotid arteries were ligated immediately following the ligation of the middle cerebral artery. The ligation sutures were removed at 1 hr from the middle cerebral and common carotid arteries. Age- and gender-matched mice were used to establish sham controls with identical procedures except for the ligation of the middle cerebral and common carotid arteries.

### Hepatocyte isolation

Hepatocytes were isolated from the liver for testing TFF3 expression by collagenase treatment and Percoll density gradient centrifugation [32,54]. Briefly, the liver of a deeply anesthetized mouse was perfused with 0.2 mg/ml collagenase in Dulbecco's modified eagle medium (DMEM) through portal vein cannulation at 30 mm Hg pressure for 20 min at 37° C. The liver was removed and mechanically dissected in culture medium to free liver cells. The resulting cell suspension was filtered through a nylon mesh (pore size 100 µm) and washed in DMEM to remove collagenase by two cycles of centrifugation at 50 g for 5 min each. To isolate hepatocytes from other cell types, 0.5 ml of cell suspension (~2 x 10^6^ cells/ml) was mixed with 1 ml of Percoll^®^ Plus (density 1.130 g/ml, Sigma-Aldrich). The mix was centrifuged at 30,000 g for 30 min at 4° C. The cell fraction on the top of the Percoll medium, containing mostly non-viable hepatocytes and non-hepatocyte cells, were removed, and the viable hepatocyte fraction located in the density range of ~1.09 g/ml was collected, washed twice in DMEM, and used for testing TFF3 expression. 

### Immunoprecipitation and immunoblot analyses for TFF3 expression

 The relative expression of TFF3 in the hepatocyte was tested by immunoprecipitation and immunoblot analyses at 0 (healthy control), 1, 3, 5, and 10 days after sham operation or cerebral ischemia/reperfusion injury. The observation period from 0 to 10 days was selected since the relative level of TFF3 expression in the hepatocyte returned to the control level at day 10 following cerebral injury (see Results section). The reason to use immunoprecipitation is that this method, together with immunoblot analysis, displays more clearly the relative expression of TFF3, which is expressed at a relatively low level under healthy conditions. This method can also be used to reduce non-specific antibody reactions [55,56]. Briefly, the isolated hepatocytes were lysed in RIPA buffer, the cell debris was removed by centrifugation, the protein concentration in the supernatant was detected by using the Bradford method, and TFF3 was isolated by immunoprecipitation with an anti-TFF3 antibody from Santa Cruz Biotechnology (1:100 antibody mixed in 250 µl of cell lysates containing about 500 µg total proteins for each sample, incubated for 12 hrs at 4° C with mild agitation). The immunoprecipitated proteins were isolated by using protein A/G agarose beads (1:20, incubated for 4 hrs at 4° C with mild agitation). The isolated proteins were reduced, resolved by electrophoresis (SDS-PAGE), transferred to a nitrocellulose membrane, and probed with an anti-TFF3 antibody. The protein bands were displayed by horseradish peroxidase-conjugated secondary antibody labeling in conjunction with a chemiluminescence method [55,56]. The relative expression of TFF3 was assessed in reference to the relative level of the protein from a healthy mouse. Each test was repeated for six times. In addition to the liver, TFF3 expression was also tested in specimens from other organs, including the ischemic brain tissue, lung, pancreas, small intestine, kidney, and skeletal muscle, at 5 days following sham operation or cerebral ischemia/reperfusion injury. The testing time at 5 days was based on the observation that the peak level of TFF3 expression was found at this time in the liver. β-actin from the same samples was tested and used as a control. 

### ELISA

 The serum level of TFF3 was measured by ELISA at day 0 (healthy control), 1, 3, 5, and 10 following sham operation or cerebral ischemia/reperfusion injury as described [32]. This observation period (from 0 to 10 days) was chosen because the relative level of TFF3 expression in the hepatocyte returned to the control level at day 10 following cerebral injury (see Results section). Serum was produced from blood samples of mice with sham operation or cerebral injury. The Invitrogen Amplex ELISA development kit with the Ultrared reagents was used for the test according to the manufacturer’s instruction. The ELISA samples were measured by using the BioTek Synergy4 plate reader. The relative serum level of TFF3 was calculated in reference to the serum level of TFF3 from healthy mice. 

### Testing the contribution of the liver to the elevation of serum TFF3

 The hepatic contribution to the elevation of serum TFF3 in cerebral ischemia/reperfusion injury was tested by partial hepatectomy immediately following the ligation of the middle cerebral and common carotid arteries. To introduce partial hepatectomy, laparotomy was carried out in the upper abdomen, and the median and left lateral lobes of the liver were ligated and removed at the common pedicle, resulting in about 60% resection of the liver [32,57]. The abdominal cavity was closed and the mouse was allowed to recover. Two sham control groups were established for the test: cerebral injury + sham hepatic operation and sham cerebral + sham hepatic operation. The partial hepatectomy model has not been shown to induce significant impairment of the hepatic functions, such as metabolism and detoxification [57,58]. The serum level of TFF3 was tested by ELISA as described above.

### Detection of neuronal degeneration by Fluoro-Jade B assay

 Degenerating neurons in the ischemic cerebral tissue were detected at 24 hrs by using the Fluoro-Jade B assay as described [59,60]. Briefly, the brain of a deeply anesthetized mouse was fixed by perfusion of 4% formaldehyde in PBS via common carotid artery cannulation at 100 mm Hg pressure and cut into 20- µm thick cryo-sections. Dried sections were rehydrated through sequential treatments with graded ethanol, treated with 0.06% KMnO_4_ solution for 20 min, incubated with an anti-MAP2 antibody for visualizing neuronal MAP2, and subsequently incubated with 0.0004% Fluoro-Jade B (Millipore) in 0.1% acetic acid solution for 30 min. Hoechst 33258 was used to stain cell nuclei. Acetic acid solution (0.1%) without Fluoro-Jade B was used as a negative control. The specimens were evaluated by fluorescence microscopy. 

### Measurement of cell death in ischemic cerebral cortex by TUNEL assay

 Cell death in the ischemic cerebral cortex was measured by the TUNEL assay. At a selected time, the brain of a deeply anesthetized mouse was fixed by perfusion of 4% formaldehyde in PBS via the left common carotid artery. Specimens were collected from the ischemic and sham control cortical cortex, and cut into 10 µm cryo-sections. The TUNEL assay was carried out by using the Roche cell death kit as described [32]. Hoechst 33258 was used to stain cell nuclei. Specimens incubated with the TUNEL reagents excluding the fluorophore-conjugated dUTP were used as negative controls. At least 6 specimen sections, equally spaced through an ischemic region, were collected from each mouse for measurements. TUNEL-positive cell nuclei were measured from 6 randomly selected regions of each specimen section. A TUNEL index was defined as the percentage of the TUNEL-positive cell nuclei in reference to Hoechst 33258-labeled total cell nuclei.

### Immunofluorescence microscopy for evaluating MAP2 organization and leukocyte infiltration to ischemic cerebrum

 The organization and distribution of microtubule-associated protein 2 (MAP2), a neuron-specific molecule, are indicative of the integrity of the neuron. Here, we visualized MAP2 in the ischemic cerebral cortex by immunofluorescence microscopy. At a selected time, the brain of mice with sham operation or cerebral ischemia/reperfusion injury was fixed by perfusion of 4% formaldehyde in PBS. Specimens were collected from the ischemic and sham control cerebral cortex, cut into 10 µm cryo-sections. The specimen sections were incubated in the presence of an anti-MAP2 antibody (Santa Cruz Biotechnology) followed by incubation with a PE-conjugated secondary antibody. Hoechst 33258 was used to label cell nuclei. MAP2 was visualized by fluorescence microscopy. 

 TFF3 is expressed in the liver in response to cerebral injury, likely by the mediation of endocrine inflammatory factors. This brings up a question whether TFF3 regulates the activity of leukocytes, which in turn influence neuroprotective processes. To address this question, we tested the density of CD45+ leukocytes within the injured cerebral cortex of wild-type and TFF3^-/-^ mice. Brain specimen sections, as prepared above, were incubated with an anti-CD45 antibody (R&D Systems) in the presence of Hoechst 33258 for labeling cell nuclei. An IgG-B antibody was used as a negative control. The density of CD45+ cells was measured within a defined area of the ischemic cerebral cortex. Six areas were randomly selected from each brain section, and six sections equally spaced through each ischemic lesion were used for the measurement. The relative density of CD45+ cells was expressed as cells/mm^2^. 

### Evaluation of Caspase 3 activity

 Caspase 3 is commonly activated in ischemia/reperfusion injury to induce cell apoptosis. The relative activity of caspase 3 was measured in sham control and ischemic cerebral specimens by using a caspase 3 colorimetric activity assay kit (Chemicon International) as described in our previous investigation [61]. For this assay, a p-nitroaniline (pNA)-conjugated caspase 3 substrate peptide, DEVD-pNA, is applied to tissue homogenates to allow DEVD-pNA reaction with cell-derived caspase 3, resulting in pNA cleavage. The rate of pNA cleavage is dependent on the level of caspase 3. The level of pNA can be measured by spectrophotometry, indicative of the relative activity of caspase 3. At 24 hrs following cerebral ischemia/reperfusion injury or sham operation, injured or sham control cerebral specimens were removed from a deeply anesthetized mouse and homogenized in RIPA buffer. The 24-hr observation time was chosen because the maximal level of cell death was found at this time as suggested by the TUNEL assay. After the cell debris was removed by centrifugation (10,000g for 5 min), the protein concentration in the supernatant was detected by using the Bradford method. An amount of 20 µg of total proteins from each specimen was applied to the provided DEVD-pNA solution and incubated for 90 min at 37° C. Samples were tested by spectrophotometry at 410 nm. Changes in caspase 3 activity were evaluated in reference to the level of sham controls.

### Measurement of cerebral infarcts by TTC and cresyl violet assays

 The degree of cerebral infarcts was evaluated by using the 2,3,5-triphenyltetrazolium chloride (TTC) [32] and cresyl violet assay as described [62]. The TTC assay was used to test acute cerebral infarction at 24 hrs, whereas the cresyl violet assay was used to evaluate cerebral infarction at 5, 10, and 30 days after cerebral ischemia/reperfusion injury. The rationale for not using the TTC assay for chronic infarction evaluation is that leukocytes infiltrated into the cerebral lesion and glial cells generated in response to cerebral injury can be stained red with TTC, obscuring the identification of cerebral infarcts. For the TTC assay, mice were deeply anesthetized at 24 hrs following cerebral injury. The brain was removed, frozen at -80° C for 5 min, cut into ~1-mm serial coronal sections, incubated in 1% TTC in PBS for 30 min at 37° C, fixed in 4% formaldehyde in PBS for 15 min, and photographed for measuring the fraction of cerebral infarcts in reference to the total cerebral volume. 

For the cresyl violet assay, mice were deeply anesthetized at 5, 10, and 30 days following cerebral injury and the brain was perfused with 4% formaldehyde in PBS via common carotid artery cannulation at 100 mm Hg pressure and cut into serial 20-µm thick cryo-sections. The brain specimens were stained with a 1% cresyl violet acetate solution. The specimen with the maximal infarct area was selected from each mouse for measurements and analyses. The areas of the cerebral infarct and the total brain section were measured and the faction of the cerebral infarct was calculated in reference to the total area of the brain section. 

### Measurement of forelimb motor function

**Figure 1:**
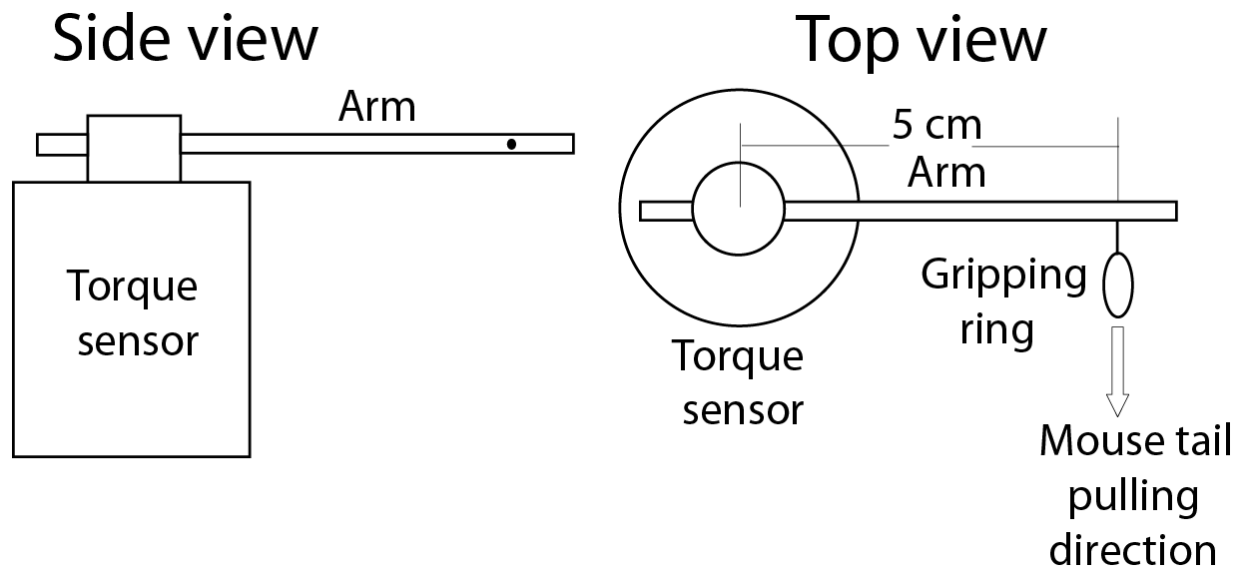
Schematic representation of the torque system used for measuring the mouse forelimb gripping strength.

 Experimental cerebral ischemia induced by ligating the middle cerebral artery induces impairment of the motor function of skeletal muscles. In this investigation, we measured and analyzed the forelimb gripping strength and forelimb wire-hanging duration to assess the degree and progression of cerebral injury in wild-type and TFF3^-/-^ mice with administration of PBS or recombinant TFF3. The forelimb gripping strength was measured by using a torque system as illustrated in Figure 1. For this test, a mouse was tail-lifted, one of the mouse forelimbs was wrapped with a piece of Scotch tape to prevent the limb from gripping, and the other forelimb was allowed to grip a small ring (1 cm) attached to the arm of a torque sensor (arm length 5 cm). The mouse was tail-pulled slowly in the direction perpendicular to the torque arm until the mouse gave up gripping. The mouse forelimb gripping force was recorded continuously during the test via a data acquisition system and used to represent the gripping strength. The other forelimb was tested alternately with the same procedure. The gripping force measurement system was calibrated by using known weights.

 The mouse forelimb motor function was also evaluated by the hanging wire test as descried [63,64]. For this test, a straight metal wire of 2 mm in diameter was suspended above a soft pillow with a wire-to-pillow distance about 8 cm. A mouse was allowed to grasp and hang on the metal wire with one of the forelimbs, when the other forelimb was wrapped with a piece of Scotch tape. The hanging duration was recorded for analyses. The other forelimb was tested alternately with the same procedure. The hanging duration was compared between the left and right forelimbs and used to analyze the forelimb motor function. 

### Administration of recombinant TFF3

 To confirm the neuroprotective action of TFF3, we administered recombinant TFF3 to TFF3^-/-^ mice with cerebral ischemia/reperfusion injury. Recombinant TFF3 (ProSpec) was administered immediately after the ligation of the middle cerebral and common carotid arteries intravenously at a dose of 50 ng/g of body weight, followed by administrations for 3 days with a 12-hr interval. PBS was used as a control. This administration strategy was established based on the observation that cell death occurs within the early 3 days following cerebral injury (see Results section). The neuroprotective effect of TFF3 was analyzed and compared between TFF3^-/-^ mice with administration of PBS and recombinant TFF3. 

### Fluorescein-TFF3 tracing test

 A fundamental question for this research is whether the naturally expressed or intravenously administered TFF3 can pass through the blood brain barrier and reach the injured cells in the ischemic brain tissue. We carried out a fluorescein-TFF3 tracing test to address this question. Recombinant TFF3 was conjugated with fluorescein by using a Roche fluorescein protein labeling kit based on the manufacturer’s instruction. Fluorescein-conjugated TFF3 (50 ng/g of body weight) was administered to mice intravenously at 24 hrs after cerebral ischemia/reperfusion injury. At 1 hr following fluorescein-TFF3 administration, the vasculature was perfused with PBS via carotid artery cannulation to remove circulating fluorescein-TFF3 while the vena cava was cut for draining blood. The brain was removed, cut into 1 mm slices, and used to identify cerebral infarcts by using the TTC assay [32] and visualize fluorescein-TFF3 in the ischemic brain tissue by fluorescence microscopy.

### Statistics

 Means and standard deviations were calculated for each measured parameter. The two-sided Student’s t-test was used for analyzing differences between two groups and the analysis of variance (ANOVA) was used for comparisons among more than two groups. Sample size in each group was estimated by power analyses. A difference was considered statistically significant at p < 0.05.

## Results

### TFF3 upregulation in hepatocytes following cerebral ischemia/reperfusion injury

**Figure 2:**
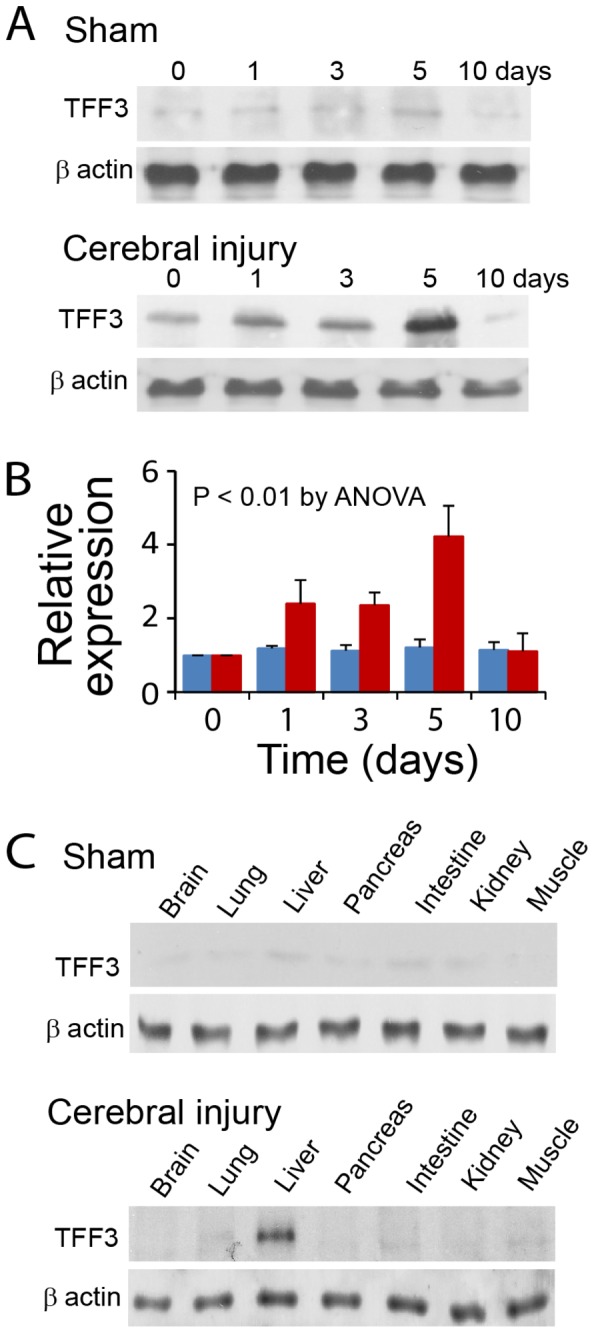
Relative expression of TFF3 in hepatocytes. (**A**) Immunoblot analyses of TFF3 expression in hepatocytes from mice with sham operation or cerebral ischemia/reperfusion injury. (**B**) Graphic representation of TFF3 expression in hepatocytes. Blue: Sham controls. Red: Cerebral ischemia/reperfusion injury. Data at time zero were from healthy mice. Relative expression was calculated in reference to the data at time zero. P < 0.001 between sham controls and cerebral injury at day 1, 3, 5, and 10 days by ANOVA (n = 6). (**C**) Immunoblot analyses of TFF3 expression in the brain, lung, liver, pancreas, small intestine, kidney, and skeletal muscle of mice with sham operation or cerebral ischemia/reperfusion injury at day 5, at which the maximal level of TFF3 expression was found in the hepatocyte.

 Cerebral ischemia/reperfusion injury induced upregulation of TFF3 expression in hepatocytes. Immunoprecipitation and immunoblot analyses demonstrated a time-dependent TFF3 expression after cerebral injury with a peak level at 5 days compared to the sham control level (Figure 2A, B). The relative level of TFF3 expression returned to the sham control level at day 10 after cerebral injury. Other selected organs, including the ischemic brain tissue, lung, pancreas, small intestine, kidney, and skeletal muscle, did not exhibit noticeable TFF3 upregulation in cerebral ischemia/reperfusion injury at the TFF3 peak expression time in the liver (Figure 2C). These observations suggested that cerebral ischemia/reperfusion injury induced a hepatic response involving upregulation of TFF3 expression. 

### Contribution of the liver to serum elevation of TFF3

**Figure 3:**
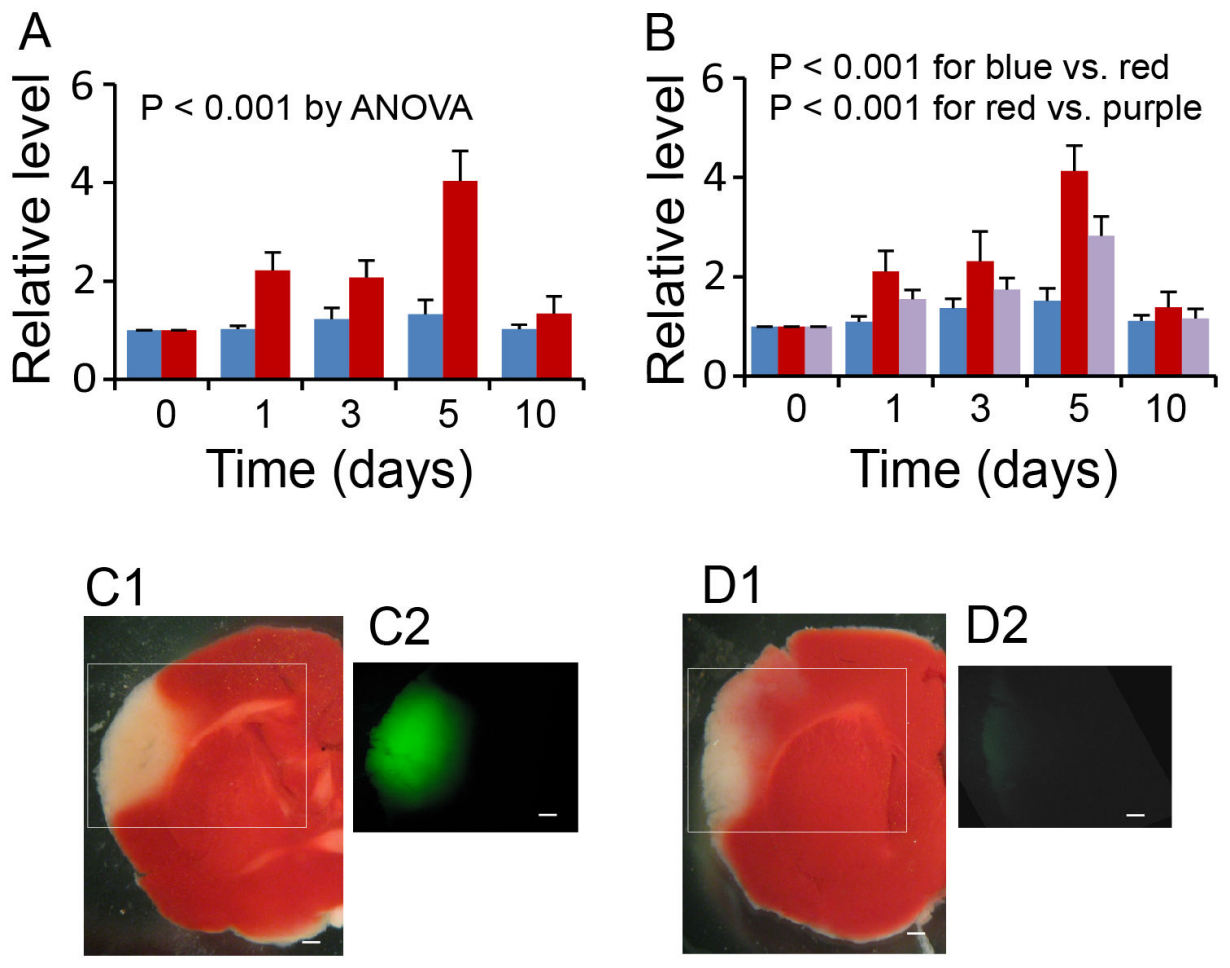
Relative serum levels of TFF3 in mice with sham operation and cerebral ischemia/reperfusion injury, and contribution of the liver to serum TFF3. (**A**) Relative serum level of TFF3 by ELISA. Blue: Sham controls. Red: Cerebral ischemia/reperfusion injury. P<0.001 between sham controls and cerebral ischemia/reperfusion injury at day 1, 3, 5, and 10 days by ANOVA (n = 8). (**B**) Contribution of the liver to serum elevation of TFF3 in cerebral ischemia/reperfusion injury. Blue: Controls with cerebral and hepatic sham operation. Red: Cerebral ischemia/reperfusion injury with hepatic sham operation. Purple: Cerebral ischemia/reperfusion injury with partial hepatectomy. P<0.001 between cerebral/hepatic sham operation and cerebral injury with hepatic sham operation, and P<0.001 between cerebral injury with hepatic sham operation and cerebral injury with partial hepatectomy at day 1, 3, 5, and 10 days by ANOVA (n = 8). For both panel A and B, data at time zero were from healthy mice. The relative level was calculated in reference to the data at time zero. (**C**) Presence of fluorescein-TFF3 in the ischemic cerebral lesion, but not in the intact brain, at 1 hr following intravenous administration of fluorescein-TFF3 to a mouse with 24-hr cerebral ischemia/reperfusion injury. C1: A TTC-stained section of the injured cerebral hemisphere showing a cerebral infarct (white) and intact cerebrum (red). C2: a fluorescence micrograph from the selected area of panel C1, showing fluorescein-TFF3 (green) in the cerebral infarct, but not in the intact cerebrum. Scale: 1 mm. (**D**) Absence of fluorescence in the ischemic cerebral lesion at 1 hr following intravenous administration of non-conjugated TFF3 to a mouse with 24-hr cerebral ischemia/reperfusion injury. D1: A TTC-stained section of the injured cerebral hemisphere showing a cerebral infarct (white) and intact cerebrum (red). D2: a fluorescence micrograph from the selected area of panel D1, showing the lack of green fluorescence in the ischemic cerebral lesion. Scale: 1 mm.

 In association with TFF3 upregulation in the hepatocyte, the serum level of TFF3 was increased significantly from day 1 to 5 after cerebral ischemia/reperfusion injury as tested by ELISA (Figure 3A). The trend of change in serum TFF3 was similar to that in TFF3 expression in the hepatocyte, suggesting a contribution of the liver to the elevation of serum TFF3. To confirm the hepatic contribution, we tested the serum level of TFF3 in the presence of cerebral ischemia/reperfusion injury and partial hepatectomy (~60% liver resection), a model known to reduce the serum level of liver-secreted proteins [32]. As shown in Figure 3B, partial hepatectomy induced a significant drop of the relative level of serum TFF3 after cerebral injury in reference to the serum TFF3 level in mice with cerebral injury + sham hepatic operation. These observations supported the contribution of the liver to the elevation of serum TFF3 in cerebral ischemia/reperfusion injury.

### Access of TFF3 to the ischemic brain tissue

 We carried out a fluorescein-TFF3 tracing test to demonstrate the access of circulating TFF3 to the ischemic brain tissue. When fluorescein-TFF3 (50 ng/g of body weight) was administered intravenously to mice with 24-hr cerebral ischemia/reperfusion injury, fluorescein-TFF3 extravasation was found in the ischemic brain tissue, but not in the intact brain tissue, within 1 hr following administration (Figure 3C). In contrast, mice with intravenous administration of non-conjugated TFF3 did not show fluorescence within the ischemic brain tissue (Figure 3D). These observations demonstrate an increase in the permeability of the blood brain barrier in the ischemic brain tissue, allowing access of circulating TFF3 to the injured brain tissue. 

### Neuronal degeneration

**Figure 4:**
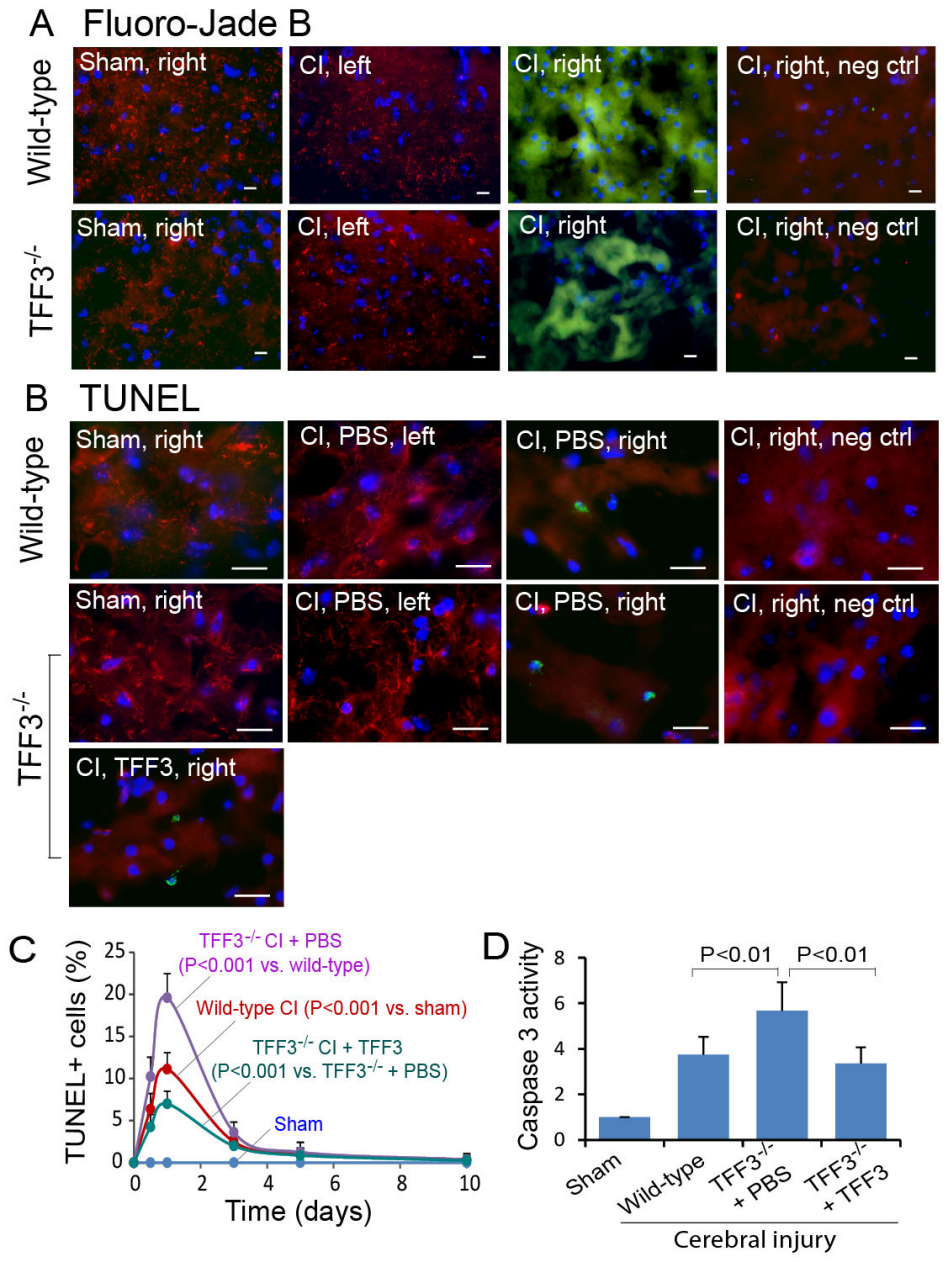
Influence of TFF3 on neuronal degeneration, cell death, and caspase 3 activity in the ischemic cerebral lesion. (**A**) Fluorescence micrographs showing Fluoro-Jade B-labeled degenerating cortical neurons in wild-type and TFF3^-/-^ mice with cerebral ischemia/reperfusion injury at 24 hrs. No Fluoro-Jade B-labeled cells were found in the cerebrum of sham control mice and the non-injured left hemisphere of the mice with right hemisphere injury. CI: Cerebral ischemia/reperfusion injury. Left: Left hemisphere. Right: Right hemisphere. neg ctrl: Negative control for Fluoro-Jade B staining (without Fluoro-Jade B). Red: MAP2. Green: Fluoro-Jade B (note that the yellowish color represents overlay of Fluoro-Jade B and MAP2 signals). Blue: cell nuclei. Scale bars: 10 µm. (**B**) TUNEL-labeled cells in the cerebral cortex of wild-type and TFF3^-/-^ mice with cerebral injury at 24 hrs. The abbreviations are the same as those for panel A, except that neg ctrl is TUNEL-negative controls (specimens incubated with TUNEL reagents excluding fluorophore-conjugated dUTP). No TUNEL-positive cells were found in the cerebrum of sham control mice and the non-injured left hemisphere of the mice with right hemisphere injury. PBS and TFF3: Intravenous administration of PBS or TFF3 immediately after cerebral injury, respectively. Red: MAP2. Green: TUNEL. Blue: cell nuclei. Scale bars: 10 µm. (**C**) Graphic representation of the density of TUNEL-positive cells in the cerebral cortex of wild-type mice with sham operation (blue) or cerebral ischemia/reperfusion injury (red) and TFF3^-/-^ mice with cerebral ischemia/reperfusion injury with administration of PBS (purple) or recombinant TFF3 (green). CI: Cerebral ischemia/reperfusion injury. P<0.001 between sham operation (blue) and cerebral ischemia/reperfusion injury (red) in wild-type mice, P<0.001 between wild-type (red) and TFF3^-/-^ (purple) mice with cerebral ischemia/reperfusion injury, and P<0.001 between cerebrum-injured TFF3^-/-^ mice with PBS (purple) and recombinant TFF3 (green) at day 1, 3, 5, and 10 days by ANOVA (n = 8). (**D**) Relative activity of caspase 3 in the ischemic cerebral lesion at 24 hrs, calculated in reference to the relative activity of caspase 3 in the sham control cerebrum.

 Neuronal degeneration occurs in cerebral ischemia. We tested neuronal degeneration by the Fluoro-Jade B assay [59,60]. In both wild-type and TFF3^-/-^ mice, almost all cerebral cells, including neurons and glial cells, within the ischemic cerebral tissue were positively labeled with Fluoro-Jade B (Figure 4A). This observation suggests that the Fluoro-Jade assay is a sensitive method for detecting degenerating neurons. In the cerebral ischemia/reperfusion model with severe neuronal infarction, this assay may not be used to identify differences between the wild-type and TFF3^-/-^ mice. 

### Alleviation of cell death by TFF3

 Cerebral ischemia/reperfusion injury caused time-dependent cell death in the ischemic cerebral tissue. Cell death was observed at 12 hrs, reached the maximal level at 1 day, and diminished at day 3 after cerebral injury as tested by the TUNEL assay, while no cell death was found in the cerebral tissue of sham control mice (Figure 4B, 4C). TFF3^-/-^ mice exhibited a significant increase in the fraction of TUNEL-positive cells most notably at 1 day compared to wild-type mice with cerebral injury (Figure 4C). Intravenous administration of recombinant TFF3 reversed the change due to TFF3 deficiency (Figure 4C), suggesting a neuroprotective role for TFF3 in cerebral ischemia/reperfusion injury. 

### Attenuation of caspase 3 activity by TFF3

Activation of caspase 3 is a key process of cell apoptosis [65,66]. As ischemia/reperfusion injury causes cell apoptosis, we evaluated the relative activity of caspase 3 in sham control and ischemic cerebral specimens from wild-type and TFF3^-/-^ mice at 24 hrs, a time chosen because of the presence of maximal cell death as tested by the TUNEL assay. Cerebral ischemia/reperfusion injury caused a significant increase in the relative activity of caspase 3 in the ischemic cerebral tissue compared to the sham control level (Figure 4D). TFF3^-/-^ mice exhibited a significant increase in the relative activity of caspase 3 in the ischemic cerebral tissue compared to wild-type mice, whereas administration of recombinant TFF3 reversed the change due to TFF3 deficiency (Figure 4D). These investigations suggested an involvement of caspase 3 in cerebral injury and a role for TFF3 in suppressing caspase 3 activation. 

### Alterations in MAP2 organization and distribution

 The organization of microtubule-associated protein 2 (MAP2) is indicative of the integrity of the neuron. In healthy neurons, MAP2 is organized into filaments and speckles (Figure 4B). In cerebral ischemia/reperfusion injury, neuronal MAP2 filaments and speckles were disassembled within 24 hrs, and MAP2 was dispersed uniformly throughout the cytoplasm (Figure 4B). Wild-type and TFF3^-/-^ mice exhibited similar changes in the pattern and distribution of MAP2 in cerebral ischemia/reperfusion injury (Figure 4B). Administration of recombinant TFF3, although mitigating cell death in the ischemic cerebral cortex, did not noticeably improve the organization of MAP2 compared to PBS administration in TFF3^-/-^ mice with cerebral injury (Figure 4B). These observations suggested that the organization of neuronal MAP2 was sensitive to ischemia/reperfusion injury, and injured neurons with disassembled MAP2 could still be rescued by administration of recombinant TFF3. 

### Influence of TFF3 on leukocyte infiltration

**Figure 5:**
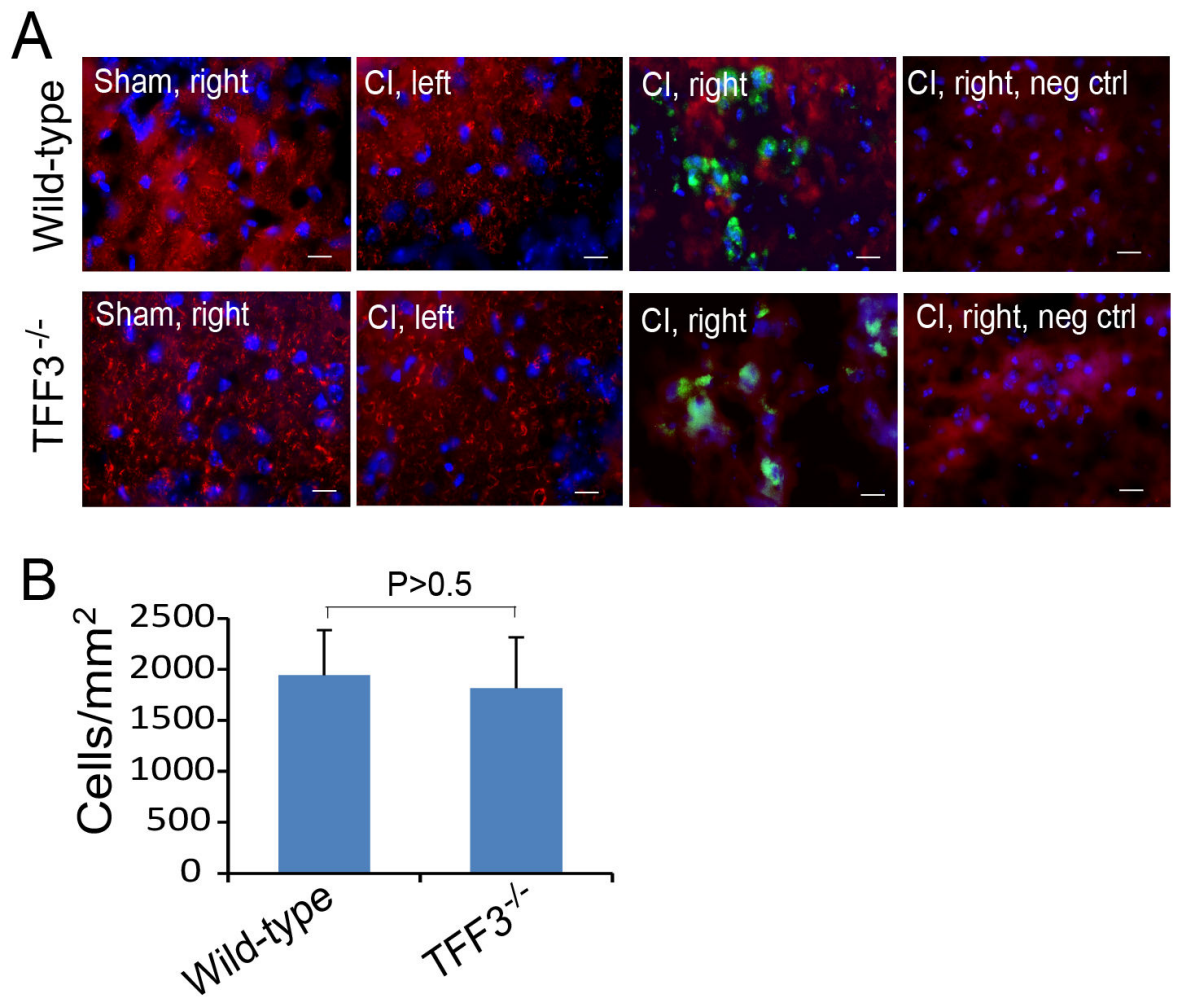
Influence of TFF3 on leukocyte infiltration into the ischemic cerebral lesion. (**A**) Fluorescence micrographs showing CD45+ leukocytes in the ischemic cerebral cortex in wild-type and TFF3^-/-^ mice. No CD45+ cells were found in the cerebrum of sham control mice and the non-injured left hemisphere of the mice with right hemisphere injury. CI: Cerebral ischemia/reperfusion injury. Left: Left hemisphere. Right: Right hemisphere. neg ctrl: Negative control for CD45 (with a control antibody instead of an anti-CD45 antibody). Red: MAP2. Green: CD45. Blue: cell nuclei. Scale bars: 10 µm. (**B**) Graphic representation of leukocyte density in the ischemic cerebral lesion of wild-type and TFF3^-/-^ mice.

 Hepatic TFF3 is upregulated in response to cerebral injury, likely via the mediation of endocrine inflammatory factors. There is a possibility that TFF3 may regulate inflammatory processes such as leukocyte infiltration into the ischemic cerebral lesion, and activated leukocytes may in turn influence neuroprotective processes. To test this possibility, we measured and analyzed the density of leukocytes infiltrated into the ischemic cerebral tissue of wild-type and TFF3^-/-^ mice at 24 hrs. Both wild-type and TFF3^-/-^ mice showed a similar density of leukocytes with a statistical significance level P > 0.5 (Figure 5). This observation suggested that TFF3 did not significantly influence leukocyte infiltration into the ischemic cerebral lesion and leukocytes might not contribute significantly to the difference in the cerebral injury level between wild-type and TFF3^-/-^ mice. However, this investigation did not test the inflammatory factors from leukocytes within the cerebral lesion and was not able to rule out the potential influence of these factors on cerebral injury and protection. 

### Mitigation of cerebral infarction by TFF3

**Figure 6:**
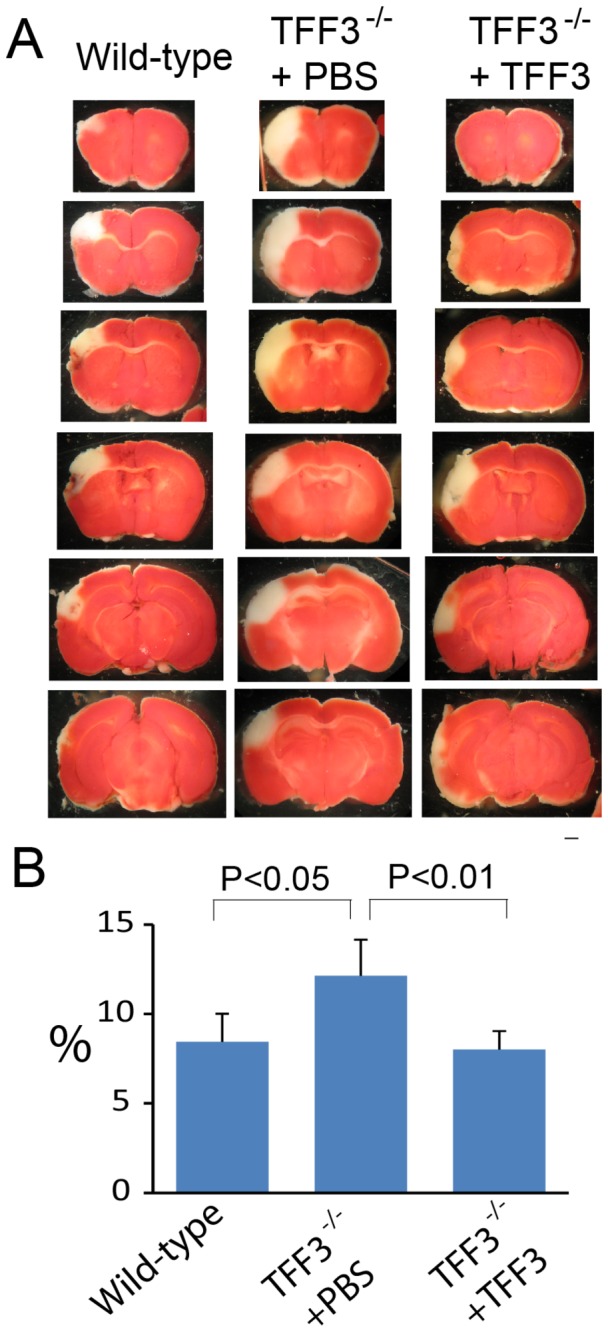
Influence of TFF3 on acute cerebral infarction. (**A**) Micrographs showing TTC-stained coronal brain slices from cerebrum-injured wild-type mice and TFF3^-/-^ mice with administration of PBS or recombinant TFF3 at 24 hrs. Scale bar: 1 mm. (**B**) Graphic representation of acute cerebral infarcts at 24 hrs in wild-type mice and TFF3^-/-^ mice with administration of PBS or recombinant TFF3.

**Figure 7:**
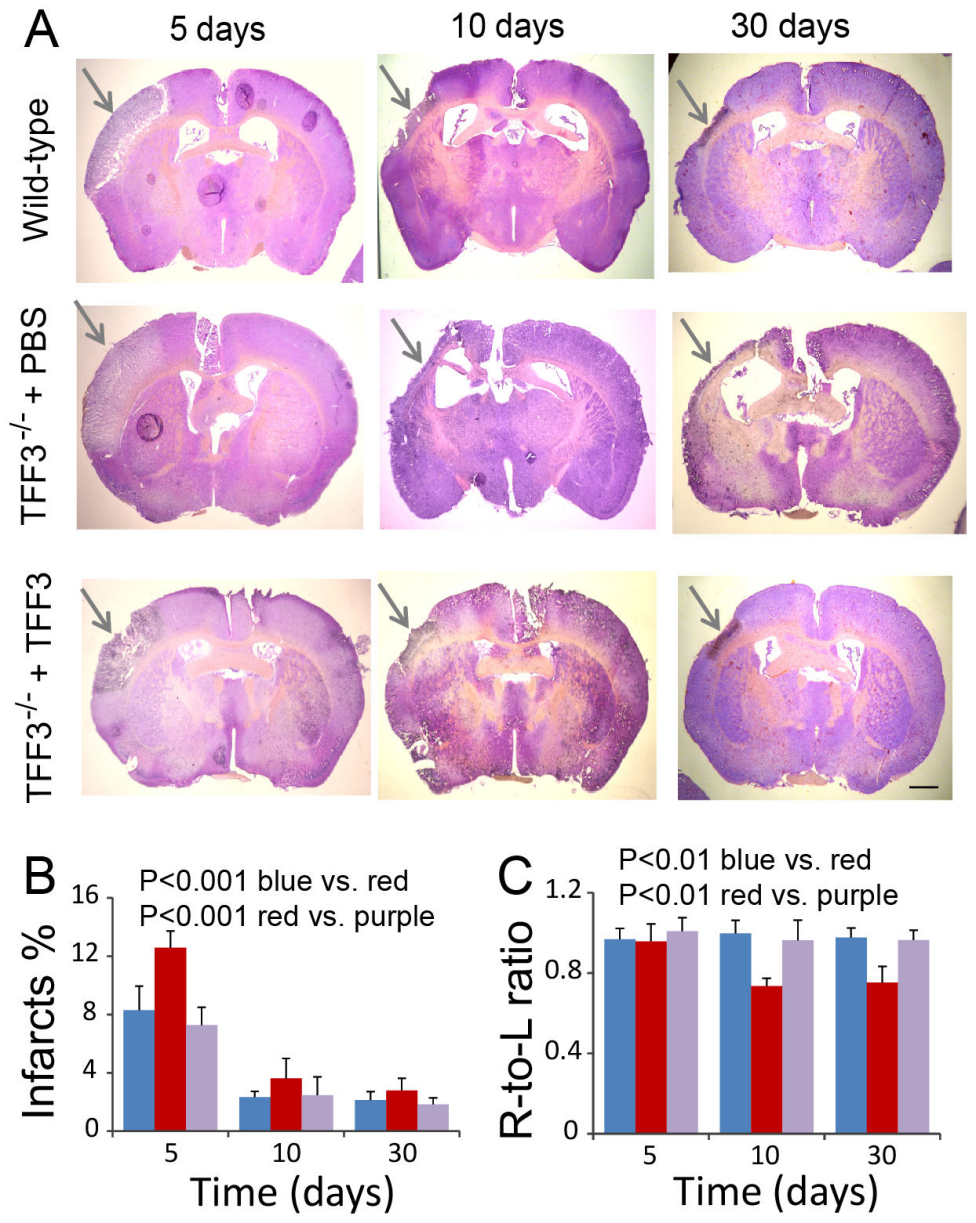
Influence of TFF3 on cerebral infarction and fibrosis. (**A**) Micrographs showing cresyl violet-stained brain specimens from wild-type mice and TFF3^-/-^ mice with administration of PBS or recombinant TFF3 at 5, 10, and 30 days after cerebral injury. Arrows: Cerebral infarcts/fibrosis. Scale bar: 1 mm for all panels. (**B**) Graphic representation of the fraction of cerebral infarcts. Blue: Wild-type mice. Red and purple: TFF3^-/-^ mice with administration of PBS or recombinant TFF3, respectively. P<0.001 between wild-type (blue) and TFF3^-/-^ mice (red) with cerebral ischemia/reperfusion injury and P<0.001 between cerebrum-injured TFF3^-/-^ mice with administration of PBS (red) and recombinant TFF3 (purple) at day 5, 10, and 30 days by ANOVA (n = 8). (**C**) Graphic representation of the ratio of the right-to-left (R-to-L) hemisphere area. The bar-color representations are the same as those for panel B. P<0.01 between wild-type (blue) and TFF3^-/-^ mice (red) with cerebral ischemia/reperfusion injury and P<0.01 between cerebrum-injured TFF3^-/-^ mice with administration of PBS (red) and recombinant TFF3 (purple) at day 5, 10, and 30 days by ANOVA (n = 8).

 Cerebral ischemia/reperfusion injury caused acute infarction in the cerebrum at 24 hrs (Figure 6), followed by fibrosis and a progressive loss of cerebral tissue in the injured hemisphere at 5, 10, and 30 days (Figure 7A, 7B). TFF3^-/-^ mice exhibited a significantly increased fraction of cerebral infarcts compared to wild-type mice at all observation times (Figures 6, 7). Furthermore, cerebral infarction was associated with a progressive loss of the total area of the injured right cerebral hemisphere (Figure 7A, 7C). TFF3^-/-^ mice exhibited a significantly smaller area of the injured right cerebral hemisphere or a smaller right-to-left hemisphere ratio at the maximal cerebral infarction site compared to wild-type mice notably at 10 and 30 days (Figure 7A, 7C). The differences in the relative area of the injured right cerebral hemisphere between TFF3^-/-^ and wild-type mice were about 26% and 23% at 10 and 30 days after cerebral injury, respectively. Administration of recombinant TFF3 to TFF3^-/-^ mice significantly reduced the fraction of cerebral infarcts and mitigated the loss of the injured cerebral hemisphere (Figure 7A, 7C). These observations suggest TFF3 as a neuroprotective protein that mitigates cerebral ischemia/reperfusion injury. 

### Improvement of forelimb motor function by TFF3

**Figure 8:**
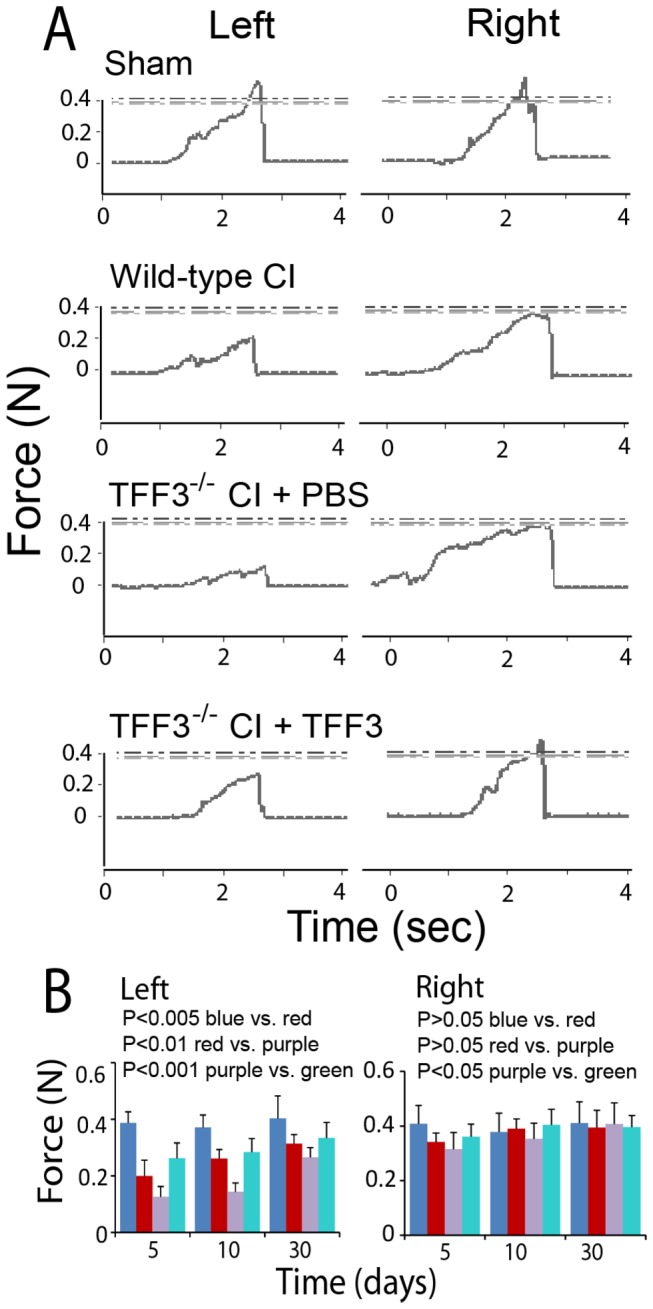
Influence of TFF3 on the forelimb gripping strength in cerebral ischemia/reperfusion injury. (**A**) Experimental records of the left and right forelimb gripping strengths from sham-operated and cerebrum-injured (CI) wild-type mice and cerebrum-injured TFF3^-/-^ mice with administration of PBS or recombinant TFF3. (**B**) Graphic representation of the gripping strengths of the left and right forelimbs. Blue and red: Wild-type mice with sham operation or cerebral injury, respectively. Purple and green: TFF3^-/-^ mice with cerebral injury with administration of PBS or recombinant TFF3, respectively. For the left forelimb, P<0.005 between wild-type mice with sham operation (blue) and cerebral injury (red), P<0.01 between wild-type (red) and TFF3^-/-^ (purple) mice with cerebral injury, and P<0.001 between cerebrum-injured TFF3^-/-^ mice with administration of PBS (purple) and recombinant TFF3 (green) at day 5, 10, and 30 days by ANOVA (n = 8). For the right forelimb, P>0.05 between wild-type mice with sham operation (blue) and cerebral injury (red), P>0.05 between wild-type (red) and TFF3^-/-^ (purple) mice with cerebral injury, and P<0.05 between cerebrum-injured TFF3^-/-^ mice with administration of PBS (purple) and recombinant TFF3 (green) at day 5, 10, and 30 days by ANOVA (n = 8).

 Right cerebral ischemia/reperfusion injury was associated with severe impairment of the left forelimb motor function and a lower degree of right forelimb motor impairment as assessed by the forelimb gripping strength test (Figure 8). The left and right forelimb gripping strengths were reduced significantly in cerebral injury compared to the sham control levels, but the left forelimb strength impairment was significantly greater than that of the right forelimb at each observation time (Figure 8). The maximal difference in the forelimb gripping strength was found at day 5 between sham operation and cerebral injury as well as between the left and right forelimbs. TFF3^-/-^ mice exhibited a significantly lowered gripping strength for both left and right forelimbs compared to wild-type mice (Figure 8). Administration of recombinant TFF3 reversed the changes in forelimb gripping strength due to TFF3 deficiency (Figure 8). These observations supported the notion that TFF3 administration improved the forelimb strength in cerebral ischemia/reperfusion injury. 

**Figure 9:**
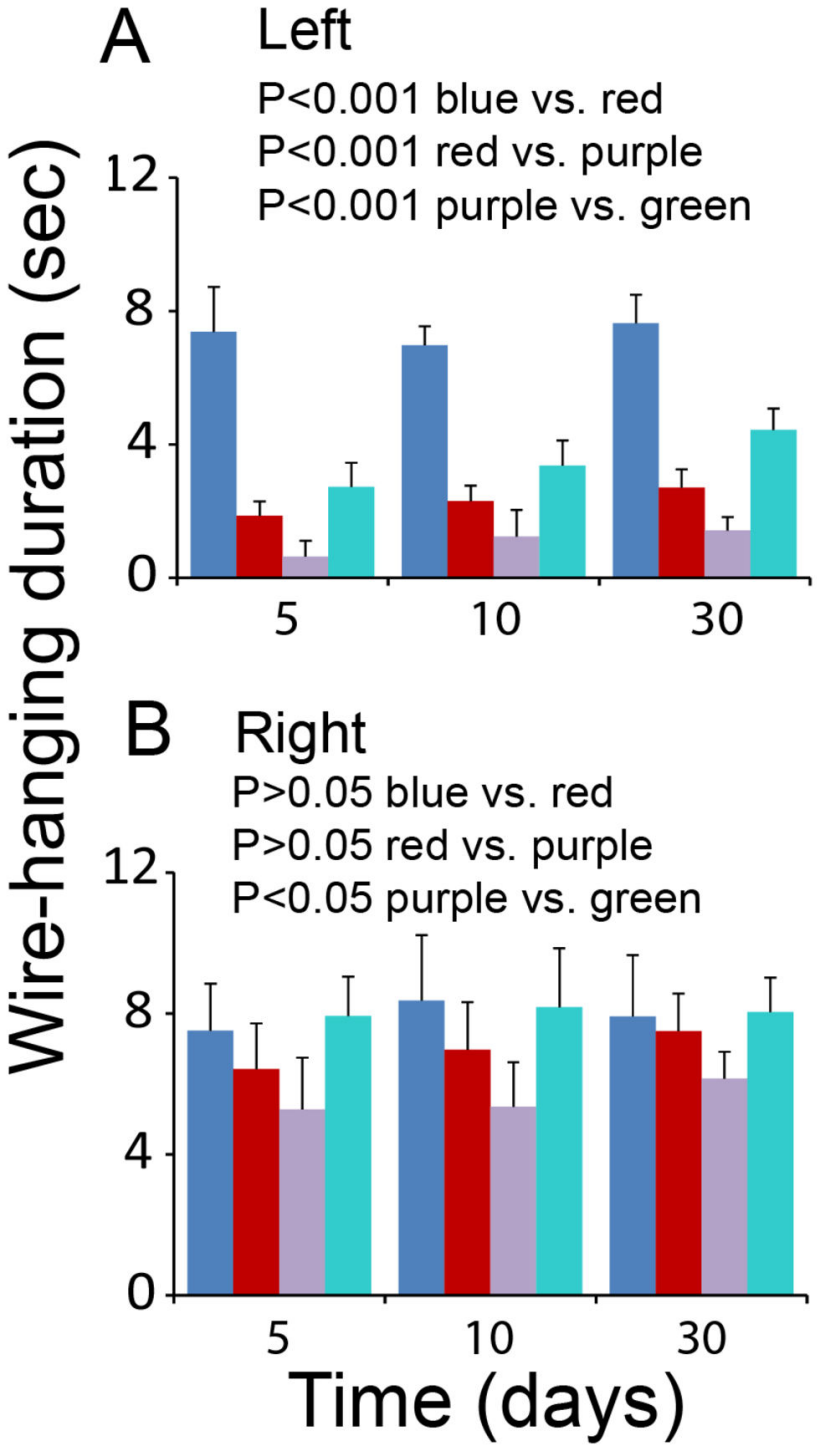
Influence of TFF3 on forelimb wire-hanging duration in cerebral ischemia/reperfusion injury. (**A**) Graphic representation of the left forelimb wire-hanging duration. Blue and red: Wild-type mice with sham operation or cerebral injury, respectively. Purple and green: Cerebrum-injured TFF3^-/-^ mice with administration of PBS or recombinant TFF3, respectively. P<0.001 between wild-type mice with sham operation (blue) and cerebral injury (red), P<0.001 between wild-type (red) and TFF3^-/-^ (purple) mice with cerebral injury, and P<0.001 between cerebrum-injured TFF3^-/-^ mice with administration of PBS (purple) and recombinant TFF3 (green) at day 5, 10, and 30 days by ANOVA (n = 8). (**B**) Graphic representation of the right forelimb wire-hanging duration. The bar-color representations are the same as those for panel A. P>0.05 between wild-type mice with sham operation (blue) and cerebral injury (red), P>0.05 between wild-type (red) and TFF3^-/-^ (purple) mice with cerebral injury, and P<0.05 between cerebrum-injured TFF3^-/-^ mice with administration of PBS (purple) and recombinant TFF3 (green) at day 5, 10, and 30 days by ANOVA (n = 8).

 We also carried out a wire-hanging duration test to confirm the beneficial effect of TFF3 on the forelimb motor function. Observations from this test were similar to those from the forelimb gripping strength test. Briefly, in right cerebral injury, the left and right forelimb wire-hanging durations were both reduced significantly compared to the sham control levels, while the left forelimb wire-hanging duration was significantly shorter than that of the right forelimb (Figure 9). TFF3^-/-^ mice exhibited a significantly shorter wire-hanging duration for both left and right forelimbs compared to wild-type mice (Figure 9). Administration of recombinant TFF3 reversed the changes in wire-hanging duration due to TFF3 deficiency (Figure 9). These observations confirmed the role of TFF3 for improving the forelimb motor function in cerebral ischemia/reperfusion injury.

## Discussion

The liver has long been considered an organ responsible for metabolism, detoxification, protein production, and bile secretion. This organ also possesses a high capacity of protection and regeneration in response to mechanical and chemical injuries [67-69]. Hepatic cells, including hepatocytes, biliary epithelial cells, endothelial cells, Kupffer cells, and Ito cells, are capable of initiating rapid proliferation after liver injury, contributing to liver regeneration [70-73]. Here, we show that the liver also contributes to neuroprotection in cerebral ischemia/reperfusion injury by upregulating and releasing the secretory protein TFF3. The neuroprotective action of TFF3 is supported by several lines of evidence, including TFF3 upregulation in the hepatocyte in response to cerebral ischemia/reperfusion injury, intensification of cerebral injury and deterioration of the forelimb motor function in TFF3^-/-^ mice, and reversal of TFF3 deficiency-induced cerebral changes by administration of recombinant TFF3 in cerebral ischemia/reperfusion injury. These findings provide a foundation for understanding the endocrine neuroprotective mechanisms and developing novel neuroprotective therapeutics. 

Hepatic upregulation of TFF3 in response to cerebral ischemia/reperfusion injury represents an endocrine protective mechanism. Such a mechanism may not be specific to cerebral ischemia/reperfusion injury. We have recently demonstrated that experimental myocardial ischemia in the mouse causes upregulation and release of secretory proteins, including TFF3 [32]. Administration of recombinant TFF3 mitigated acute myocardial infarction [32]. These observations, together with the findings from the present investigation, suggest activation of a coordinated endocrine protective mechanism involving the liver in response to an ischemic injury in a remote organ. However, it remains to be investigated whether ischemic injury in other organs causes TFF3 upregulation and activation of the liver-mediated protective mechanism. 

 There are two fundamental questions about the present investigation: how cerebral ischemia causes hepatic upregulation of TFF3 and how TFF3 protects ischemic neurons from irreversible injury. While we are not able to address these questions here because of the difficulty of approaching these problems and the lack of fundamental information in the literature, we propose the following hypothetical mechanisms for future investigations. For the first question, inflammatory mediators released from the ischemic cells, activated glial cells, and recruited leukocytes are possibly responsible for hepatic upregulation of TFF3. Several cytokines, including TNFα [74-77], IL-1 beta [74,76,77], and IL-6 [76,78,79] have been shown to participate in cerebral ischemia-induced inflammatory processes, but a complete database including all possible inflammatory mediators remains to be established in cerebral ischemia/reperfusion injury. Thus, it is necessary to test and screen all upregulated and released inflammatory mediators to identify those responsible for inducing TFF3 expression in the hepatocyte. We are currently conducting cytokine profile analyses to identify potential inflammatory mediators upregulated in response to cerebral ischemia/reperfusion injury. This approach, together with functional screening tests for the role of each inflammatory mediator in regulating hepatic expression of TFF3, will provide insights into the first question. 

For the second question, TFF3 likely interacts with cognate receptor(s) in injured neurons to activate signaling networks for neuroprotective actions. Several laboratories have intended to identify TFF3 receptors in the intestinal epithelial cell, showing that TFF3 can bind a 28-kDa protein [50] and a 50 kDa glycoprotein [51]. However, these proteins have not been identified and characterized, and their signaling mechanisms have not been tested. TFF3 has also been shown to induce phosphorylation of epidermal growth factor receptor (EGFR), which may in turn activate the cell survival signaling molecules PI3K and Akt in a colonic epithelial cell line (HT-29) and a gastric cancer cell line (AGS) [80,81]. However, these investigations did not demonstrate TFF3 binding to EGFR. It remains to be determined whether EGFR phosphorylation is a result from direct TFF3 binding or activation of other TFF3-mediated signaling events [33]. To date, a TFF3 receptor has not been identified in the neuron. We intend to identify neuronal TFF3 receptor(s) by using a TFF3 ligand–receptor crosslinking assay coupled with mass spectrometry. This approach may provide insights into the second question. 

A technical concern is whether upregulated or administered TFF3 in the circulation can pass through the blood brain barrier to access the injured cells in the ischemic cerebral lesion. Previous investigations have demonstrated that the blood brain barrier, a tight endothelium/basement membrane structure preventing lipid-insoluble molecules from entering the brain interstitial space, exhibits a rapid dramatic increase in permeability in response to ischemic brain injury, allowing diffusion of lipid-insoluble molecules and infiltration of leukocytes into the injured brain tissue [10]. The present investigation showed that fluorescein-TFF3 was able to reach the interstitial space of the ischemic cerebral lesion within 1 hr following intravenous TFF3 administration. This observation supports the approach of TFF3 administration for neuroprotection in cerebral ischemia/reperfusion injury. 

## Conclusions

The present investigation demonstrated a previously unrecognized endocrine neuroprotective mechanism involving the secretory protein TFF3 upregulated in the liver in response to cerebral ischemia/reperfusion injury. TFF3 was released into the circulation, alleviated cerebral cell death, mitigated caspase 3 activity, reduced the fraction of cerebral infarcts, and improved the motor function of the forelimbs. These findings contribute to the establishment of a new paradigm for cerebral ischemia research and development of neuroprotective therapeutics.
